# The protective effect of vinpocetine against Estradiol-benzoate induced cervical hyperkeratosis in female rats via modulation of SIRT1/Nrf2, and NLRP3 inflammasome

**DOI:** 10.1038/s41598-024-69431-2

**Published:** 2024-08-19

**Authors:** Remon R. Rofaeil, Reham H. Mohyeldin, Ehab E. Sharata, Mina Ezzat Attya, Hany Essawy, Osama A. Ibrahim, Walaa Yehia Abdelzaher

**Affiliations:** 1 Department of Medical Pharmacology, Faculty of Medicine, Minia University, Minia, 61519 Egypt; 2 Department of Pharmacology & Toxicology, Faculty of Pharmacy, Deraya University, Minia, 61111 Egypt; 3 Department of Pathology, Faculty of Medicine, Minia University, Minia, 61519 Egypt; 4 Department of Obstetrics & Gynecology, Faculty of Medicine, Minia University, Minia, 61519 Egypt

**Keywords:** Hyperkeratosis, Estradiol, Vinpocetine, NLRP3, Nrf2, Biochemistry, Cancer

## Abstract

The current study was assigned to determine the putative preventive role of vinpocetine (VIN) in cervical hyperkeratosis (CHK) in female rats. Estradiol Benzoate (EB) was utilized in a dose f (60 μg/100 g, i.m) three times/week for 4 weeks to induce cervical hyperkeratosis. VIN was administered alone in a dose of (10 mg/kg/day, orally) for 4 weeks and in the presence of EB. Levels of malondialdehyde (MDA), total nitrites (NOx), reduced glutathione (GSH), interleukin-18 (IL-18), IL-1β, tumor necrosis factor-alpha (TNF-α) were measured in cervical tissue. The expression of NLRP3/GSDMD/Caspase-1, and SIRT1/Nrf2 was determined using ELISA. Cervical histopathological examination was also done. EB significantly raised MDA, NOx, TNF-α, IL-18, IL-1β, and GSDMD and up-regulated NLRP3/Caspase-1 proteins. However, GSH, SIRT1, and Nrf2 levels were reduced in cervical tissue. VIN significantly alleviates all biochemical and histopathological abnormalities. VIN considerably mitigates EB-induced cervical hyperkeratosis via NLRP3-induced pyroptosis and SIRT1/Nrf2 signaling pathway.

## Introduction

The presence of a thicker keratin layer on the surface of stratified squamous epithelium is known as cervical hyperkeratosis (CHK)^1,2^. CHK is typically associated with inflammation, trauma, or infection, and it frequently affects women who use diaphragms^3^. A benign structural change of the cervical squamous epithelium, CHK may mask dysplastic lesions and complicate a reliable colpo-cytological examination^4^.

Estrogen strongly affects the uterine cervix^5,6^. With rising estrogen levels throughout the monthly cycle, cervical epithelial cells divide and multiply, producing hyperplastic epithelium without pathogenic alterations. High dosages or long-term use of estrogen can lead to cervical lesions as CHK^7^. Animal models exposed to estradiol benzoate (EB) displayed characteristics that might be indicative of stromal invasion and cervical cancer^3,8^. So, EB was utilized in the current study to induce CHK in female rats.

Pyroptosis is a highly inflammatory form of cell death that is closely linked to oxidative stress and the activation of the NLRP3 inflammasome. This process plays a crucial role in the initiation and amplification of inflammatory responses, with important implications for the understanding and treatment of various pathological conditions^9^.

Inflammation in the uterus is brought on by exposure to unopposed estrogen^10^. Proinflammatory cytokines and inflammatory mediators contribute to uterine hyperplastic alterations and carcinoma^11^. This can boost estrogen production and disturb the estrogen-progestogen balance, potentially leading to carcinogenesis^12^. Rapid cell division brought on by inflammation raises the concentration of free radicals, which damages DNA and induces oxidative stress^12,13^. Numerous investigations have revealed a close correlation between the inflammatory response triggered by estrogen and the activation of the NOD-like receptor family pyrin domain containing 3 (NLRP3) inflammasome, which in turn activates inflammatory mediators including IL1β, IL18, and TNF-α^14–16^. Cervical cancer is also strongly associated with up-regulation of NLRP3^17^.

The cytoprotective properties of nuclear factor (erythroid-derived 2)-like-2 factors (Nrf2) against oxidative stress and inflammation play a vital role in the removal of free radicals^18,19^. Since Nrf2 prevents normal cells from transforming, it is regarded as a trustworthy marker in cervical cancer^20,21^. Additionally, Silent mating type information regulation 2 homolog 1 (SIRT1), a member of the large Sirtuin family, regulates stress responses and cell survival through its histone deacetylase activity^22^. The expression SIRT1 is significantly correlated with endometrial cancer risk factors. Targeting SIRT1 is thought to be an effective way to treat cervical cancer^23^.

Vinpocetine (VIN) is a synthetically produced derivative of the periwinkle plant Vinca minor's alkaloid vincamine^24^. Around 1978, VIN was first created and promoted in Hungary. For the prevention and treatment of stroke, senile dementia, and memory problems, VIN has been utilized in numerous Asian and European countries. Additionally, a variety of brands of nutritional supplements with VIN in them are being offered all over the world. Previous studies have proven beyond doubt that vinpocetine has an excellent safety profile, consequently, increasing efforts have been put into exploring the novel therapeutic effects and mechanism of actions of vinpocetine in various cell types and disease models^25^. VIN demonstrated antioxidant and anti-inflammatory properties in a variety of animal studies including hepatic and renal ischemia–reperfusion injury^26,27^, neurodegeneration induced by aluminum chloride^28^, and cisplatin-induced acute kidney injury^29^. Furthermore, VIN downregulated the NLRP3 inflammasome, decreased inflammatory mediators, and provided protection against ischemic stroke^30^ and nonalcoholic steatohepatitis^31^ as well as its potential to mitigate pancreatitis via SIRT1/ Nrf2/TNF-α signaling pathway^32^. Based on these findings, it is possible to hypothesize that VIN via suppression of inflammation, might be effective in attenuating EB-induced CHK in female rats.

## Materials and methods

### Drugs and chemicals

VIN was obtained from Amirya Co., Egypt. Estradiol Benzoate (EB) powder was obtained from Misr Co., Egypt. The present study employed the highest commercially available analytical grade for all other chemical reagents.

### Animals and experimental design

Female Wistar albino rats weighing 180–210 g and aged 8–10 weeks were procured from Egypt's National Research Centre in Cairo. Before commencing the experiment, rats were acclimatized in their cages (6 rats per cage) for 1 week in a regular light–dark cycle with unrestricted access to tap water and normal food. Our experimental protocol received permission from the Study Ethics Committee of the Faculty of Medicine, Minia University (Approval number: 393:2022), and all methods were performed in accordance with the relevant scientific guidelines and regulations. The current study is reported in accordance with ARRIVE guidelines.

Twenty-four Rats were randomly divided into the following 4 groups (n = 6):

Group I (Control): rats were given carboxymethyl cellulose (CMC) orally and olive oil intramuscular injection (i.m.) three times/week for a duration of 4 weeks^3^.

Group II (VIN): rats administered VIN (10 mg/kg/day, orally)^33,34^ suspended in CMC and i.m injection of olive oil three times/week for a duration of 4 weeks.

Group III (EB): rats administered EB in a dose of (60 μg/100 g, i.m) three times/week for a duration of 4 weeks^3^.

Group IV (EB/VIN): rats received VIN (10 mg/kg/day, orally)^33,34^ plus EB in a dose of (60 μg/100 g, i.m) three times/week for a duration of 4 weeks^3^.

### Sample collection

At the close of the experiment, rats received an i.p. injection of urethane (25% in a dose of 1.6 g/kg)^35^. Rats were euthanized by cervical dislocation, and their cervices were removed and cleansed with saline to eliminate any blood. A portion was kept for histological examination. The other parts were split into two portions, the first of which was immediately frozen at -80°C until utilized for western blot analysis. For measuring biochemical parameters, the second portion was homogenised with phosphate buffer (0.01 M, pH 7.4; 20% w/v) (tissue weight (g): phosphate buffer (mL) volume = 1:5)^36^, then the homogenate was centrifuged for 15 min at 5000 rpm, and the supernatant was kept at – 80 °C.

### Biochemical investigations

#### Assessment of oxidative stress parameters

Cervical reduced glutathione (GSH); (Cat. No.: GR 25 11), and malondialdehyde (MDA); (Cat. No.: MD 25 29) were measured by kits provided by Biodiagnositic, Giza, Egypt. Total nitrite/nitrate (NOx) was determined using the Griess reaction between nitrite and a mixture of naphthyl ethylenediamine and sulfanilamide; the NO level was detected at 540 nm^37^.

#### Assessment of inflammatory markers

Cervical TNF-α (Cat. No.: E-EL-R0019), IL-18 (Cat. No.: E-EL-R0567), and IL-1β (Cat. No.: E-EL-R0012), were evaluated using ELISA kits (Elabscience, Houston, TX, USA) following the manufacturer’s guidelines.

#### Assessment of SIRT1/Nrf2 signaling pathway

Cervical SIRT1; (Cat. No.: MBS2600246), and Nrf2; (Cat. No.: MBS012148) were measured using ELISA kits provided by MyBioSource, San Diego, California, USA according to the manufacturer’s guidelines.

#### Assessment of NLRP3/GSDMD/Caspase 1 signaling pathway

Cervical NLRP3 (Cat. No.: ab277086, Abcam, UK), GSDMD; (Cat. No.: MBS2705517, MyBioSource, San Diego, California, USA), and caspase 1 (Cat. No.: E-EL-R0371, Elabscience, Houston, TX, USA) were evaluated using ELISA kits following the manufacturer’s guidelines.

### Histopathological study

The cervices of the female rats were removed, processed, and embedded in paraffin after being submerged in a 10% formalin solution for 24 h. With a microtome, cross-sections 5 μm thick were cut. Hematoxylin–eosin stain was applied to these tissue sections, and they were examined using an Olympus light microscope for a histological assessment.

### Statistical analysis

The mean ± SEM was used to present all the data. The Tukey-Kramar test was conducted after a one-way analysis of variance (ANOVA) was used to examine the data. Significant P values were defined as those less than 0.05. The statistical analysis was carried out with GraphPad Prism^®^, Version 10.00 for Windows.

## Results

### Impact of VIN on oxidative stress parameters in cervical tissue

As presented in Table 1, EB significantly increased MDA and NO and reduced GSH, as compared to control group. On the other hand, VIN when co-administered with EB, significantly reduced both MDA and NOx and increased GSH, relative to EB groups.

**Table 1:** Impact of VIN on oxidative stress parameters.

Groups	Cervical MDA (nmol/g tissue)	Cervical GSH (nmol/g tissue)	Cervical NOx (µmol/g tissue)
Control	12.1 ± 0.68	54.3 ± 2.52	47.7 ± 2.82
VIN	11.7 ± 0.73	51.8 ± 1.53	48.9 ± 1.80
EB	35.7 ± 2.33^###^	28.9 ± 2.42^###^	100 ± 3.26^###^
EB/VIN	12.1 ± 0.85***	53.0 ± 2.55***	51.0 ± 3.96***

Results represent the mean ± SEM (n = 6), *where *^***###***^*p* < 0.001, relative to control group, and ***p < 0.001 *relative to EB group.* VIN; Vinpocetine, EB; Estradiol-benzoate.

### Impact of VIN on cervical inflammatory markers

**Figure 1:**
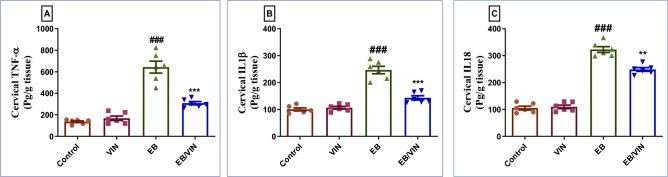
Cervical tissue levels of TNF-α (**A**), IL1β (**B**), and IL18 (**C**). Data are represented as mean ± SEM (n = 6). Where ^###^*p* < 0.001, relative to control group, **p < 0.01 relative to EB group and ***p < 0.001 relative to EB group. VIN; Vinpocetine, EB; Estradiol-benzoate, TNF-α; Tumor necrosis factor-alpha, IL18; Interleukin 18, and IL1β; Interleukin 1β.

In group received EB, TNF-α, IL-18, and IL-1β were increased significantly, relative to control group. In contrast, in EB/VIN group, a significant reduction in TNF-α, IL-18, and IL-1β occurred, as compared to EB group (Fig. 1).

### Impact of VIN on cervical SIRT1/Nrf2 signaling pathway

**Figure 2:**
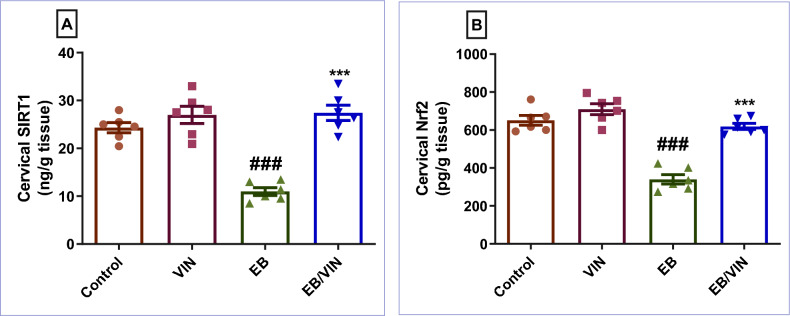
Cervical tissue levels of SIRT1 (**A**), and Nrf2 (**B**). Data are represented as mean ± SEM (n = 6). Where ^**###**^*p* < 0.001, relative to control group, and ***p < 0.001 relative to EB group. VIN; vinpocetine, EB; Estradiol-benzoate, SIRT1; Silent mating type information regulation 2 homolog 1, and Nrf2; Nuclear factor erythroid 2-related factor 2.

As presented in Fig. 2, EB significantly reduced SIRT1 and Nrf2, relative to control group. However, VIN when co-administered with EB, significantly reversed the condition as it increased both SIRT1 and Nrf2, as compared to EB group.

### Impact of VIN on cervical NLRP3/GSDMD/Caspase 1 signaling pathway

**Figure 3:**
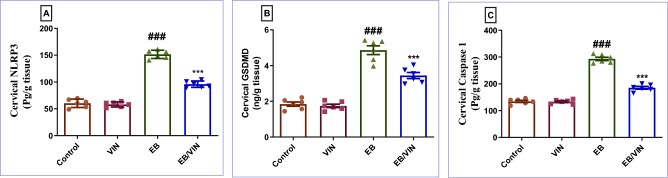
Cervical tissue levels of NLRP3 (**A**), GSDMD (**B**), and Caspase 1 (**C**). Data are represented as mean ± SEM (n = 6). Where ^###^*p* < 0.001, relative to control group, and ***p < 0.001 relative to EB group. VIN; vinpocetine, EB; Estradiol-benzoate, and NLRP3 = NOD-like receptor family pyrin domain containing 3.

In the EB group, NLRP3, GSDMD, and caspase-1 levels were all considerably increased, in relation to the control group. In contrast to EB group, In EB/VIN group, a significant decrease was observed in NLRP3, GSDMD, and caspase 1 (Fig. 3).

### Histopathological results

**Figure 4:**
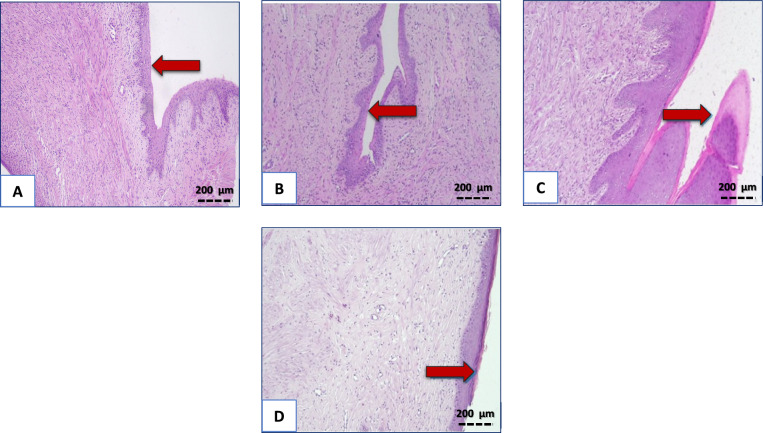
Impact of VIN on histopathological picture of rats’ cervices. Control group, and VIN group (**A**,**B**) displaying normal cervices lined by stratified squamous non keratinized epithelium. EB group (**C**) showing diffuse and marked cervical hyperkeratosis. EB + VIN group (**D**) displaying only mild and focal areas of hyperkeratosis, (×200). EB; Estradiol-benzoate, VIN; Vinpocetine**.**

Sections examined from control and VIN groups showed normal cervices lined by stratified squamous non keratinized epithelium. In contrast, cervices of group received EB showed marked and diffuse areas of hyperkeratosis. On the other hand, group in which VIN was co-administered with EB, only mild focal areas of hyperkeratosis were noticed (Fig. 4).

## Discussion

CHK is a prevalent gynecological condition caused by excessive administration of estrogen^5^.

Over-administration of estrogen contributes significantly to CHK and cervical cancer by inducing cytokines, oxidative stress, and the generation of free radicals^10^. Estrogen administration, particularly when combined with other substantial risk factors such as multiparity and human papillomavirus (HPV) infection, can lead to cervical cancer^38^. In the current study, EB used as a positive control drug that induced CHK as mentioned in previous studies and confirmed by a typical histopathological alterations^3,39^. The development of CHK is heavily influenced by oxidative stress^3^. We investigated oxidative stress markers such as MDA, NOX, and GSH to assess the oxidative stress effect of EB therapy. The negative impacts of EB were demonstrated by a considerable rise in cervical MDA, and NOx, as well as a significant decrease in cervical GSH relative to the control group. Co-administration of VIN with EB in the current study significantly improved oxidative stress status as shown by reduction in MDA, NOx and increase in GSH. This result indicates an antioxidant effect of vinpocetine in CHK. This is also documented by recent Fattori et al., study showing that vinpocetine has a considerable oxidative stress ameliorating effect^40^.

Earlier studies demonstrated that EB administration can trigger CHK by stimulating inflammation and releasing inflammatory mediators such as TNF-α, and IL1β^3,41,42^.Our study showed a significant rise in TNF-α, IL18, and IL1β levels in the EB group. Fortunately, VIN significantly reduced TNF-α, IL18, and IL1β levels, demonstrating its protective effect against EB-induced CHK, which is in line with previous studies that reported the antioxidant, and anti-inflammatory properties of VIN in several animal models, including acute kidney injury, lung inflammation caused by lipopolysaccharide, otitis media in mice, and inflammatory pain^43–45^.

To acquire a better understanding of the mechanism of VIN's protective impact against EB-induced CHK, NLRP3, Caspase-1, and GSDMD levels were investigated. NLRP3 (nucleotide-binding oligomerization domain receptors) are intracellular proteins that play a function in mammalian immunity and are highly expressed in cervical carcinoma^17^. To form an inflammasome complex, NLRP3 binds to ASC and subsequently activates procaspase 1. Mature IL-1β and IL-18 are produced from pro-IL-1β and pro-IL-18 by active caspase1, However, caspase1 also encourages GSDMD to become cleaved GSDMD, which causes the plasma membrane to open up significantly and starts the process of pyroptosis^46^. Our study showed a significant rise in cervical NLRP3, Caspase-1, and GSDMD levels in the EB group demonstrating that NLRP3 inflammasome is strongly associated with CHK. Conversely, VIN resulted in a markedly reduced expression of NLRP3, Caspase-1, and GSDMD. This is consistent with the findings of Dong Han et al. (2020), whereby the NLRP3 signaling pathway was suggested as a plausible explanation for VIN's ability to mitigate ischemic stroke^47^.

In the same vein, EB injection induced histological alterations, characterized by prominent hyperkeratosis with a thicker keratin layer on the surface of stratified squamous epithelium with underlying significant stromal inflammatory cell infiltration. These findings are in line with previous studies^48,49^. In EB/VIN group, there is marked improvement of these histopathological alterations. As only mild focal areas of hyperkeratosis were noticed. Additionally, the improvement of histopathological aberrations was supported by the downregulation of cervical inflammatory cytokines, NLRP3, caspase 1, and oxidative stress markers.

To provide more insight into the potential protective mechanism of VIN against EB-induced CHK, an evaluation of the SIRT1/Nrf2 signaling pathway was conducted. As many of its downstream target genes and enzymes are in charge of avoiding or reversing intracellular redox imbalances, Nrf2 is regarded as a master regulator of the antioxidant response^50,51^. Well-known stress response protein SIRT1 is essential for a variety of cellular and physiological processes including cell damage, and mitochondrial biogenesis and its expression is correlated with endometrial cancer^23,52^. Moreover, some investigations have suggested that SIRT1 may activate Nrf2 in order to exhibit its antioxidative actions^53,54^. Earlier studies demonstrated that downregulating Nrf2 is associated with NLRP3 activation and release of inflammatory mediators supporting the connection between the two investigated pathways in the current work^55,56^. According to the results of the current investigation, the SIRT1 and Nrf2 levels were lower in the EB-treated group. Inversely, VIN increased SIRT1 and Nrf2 expression. Additionally, these results are consistent with previous research showing the stimulatory effect of VIN on SIRT1/Nrf2 in acute pancreatitis produced by l-arginine^32^.

## Conclusion

**Figure 5:**
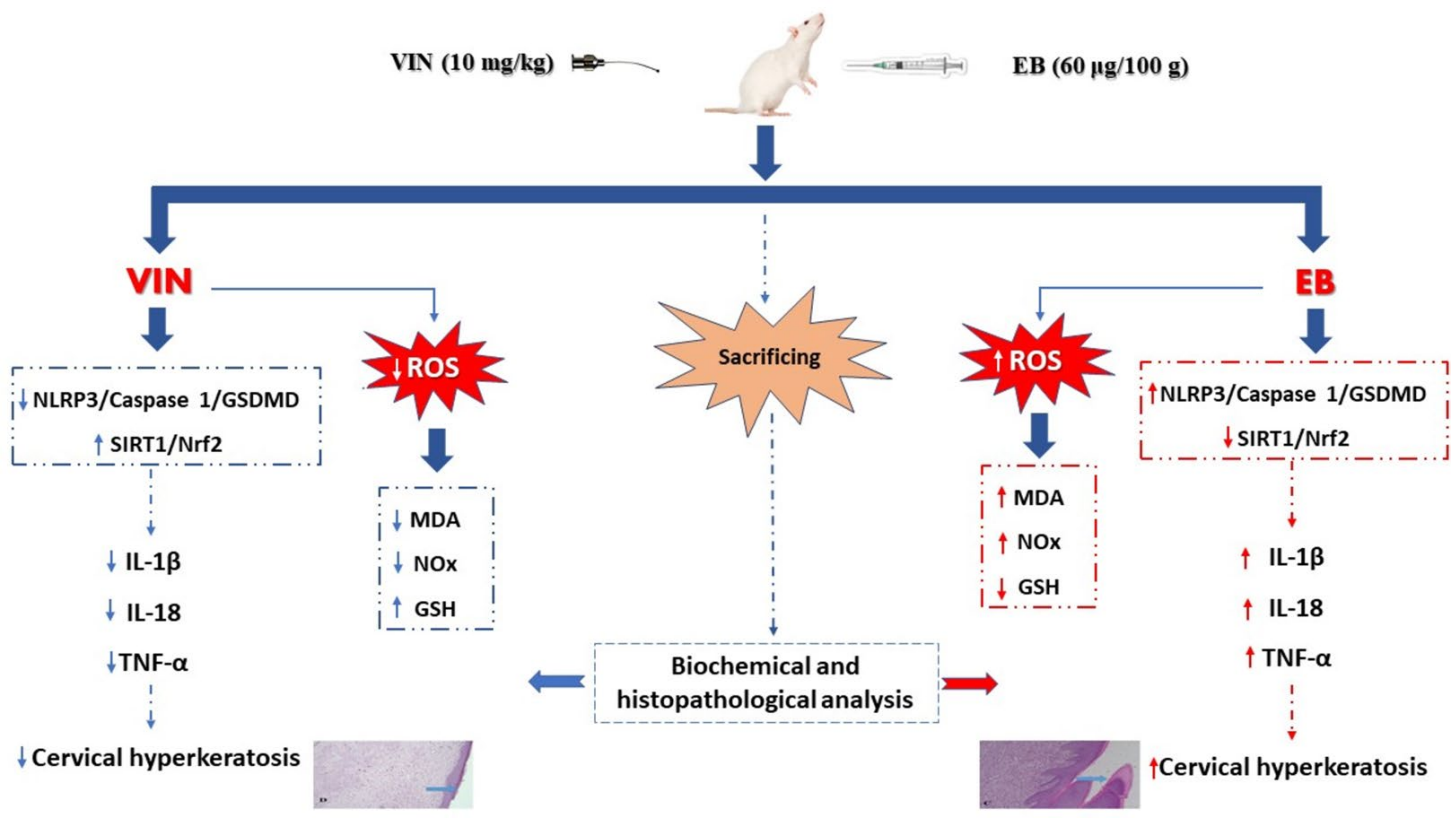
Graph outlining the mechanism of EB-induced CHK and the potential protective effect of VIN. One of the authors, Ehab E. Sharata, used Microsoft PowerPoint to create this graph.

In the current study, collectively, VIN antagonized oxidative stress, and inflammation induced by EB as evidenced by reduction in MDA, NOx, TNF-α, IL18, and IL1β with increase in GSH and via stimulation of SIRT1/Nrf2 signaling pathway and subsequent inhibition of NLRP3/Caspase 1/ GSDMD signaling pathway as illustrated in Fig. 5.

## Data Availability

The datasets used and/or analysed during the current study are available from the corresponding author on reasonable request.
